# Supplementary material for: A Systematic Review of Community-Based Exercise Interventions for Adults with Intellectual Disabilities
Source: Healthcare (Basel). 2025 Feb 1;13(3):299. doi: 10.3390/healthcare13030299 (PMC11817475; doi:10.3390/healthcare13030299)
Supplement: Supplementary file 1 [file healthcare-13-00299-s001.zip › healthcare-3361810-supplementary.pdf]

OVID (Medline)

Search	Keyword
**1**	Exp Learning Disorders/
**2**	Learning disab*
**3**	Exp Intellectual Disability
**4**	Mental* retard*.mp
**5**	Intellectual* disab*.mp
**6**	Exp Developmental Disabilities/
**7**	Developmental* disab*.mp
**8**	Mental* handicap*.mp
**9**	Mental* disab*.mp
**10**	1 or 2 or 3 or 4 or 5 or 6 or 7 or 8or 9
**11**	Exp Motor Activity/
**12**	Physical* activ*.mp
**13**	Exp Exercise/
**14**	Exercis*.mp
**15**	Exp Physical fitness/
**16**	Physical* fit*.mp
**17**	Exp Health Promotion/
**18**	Health promot*.mp
**19**	11 or 12 or 13 or 14 or 15 or 16 or 17 or 18
**20**	10 and 19
**21**	Limit 20 to (yr="1995-Current" and "all adult (19 plus years)")

EMBASE

**1**	Exp learning disorder/
**2**	Intellectual impairment/
**3**	Exp mental deficiency/
**4**	Exp developmental disorder
**5**	((mental$ or intell$) adj3 (impair$ or retard$ or defici$ or handicap$ or subnormal$)).tw
**6**	(learning$ adj (impair$ or disab$)).mp
**7**	1 OR 2 OR 3 OR 4 OR 5 OR 6
**8**	Exp exercise/
**9**	Exp physical activity/
**10**	health promotion/
**11**	(exercis$ or physical$ activ$ or leisure centre$ or gym$ or health promot$).tw
**12**	8 OR 9 OR 10 OR 11
**13**	7 AND 12
**14**	Limit 13 to (english language and yr="1995 – Currrent" and (adult <18 to 64 years> or aged <65+ years>))

CINAHL

**S1**	(MH "Intellectual Disability")
**S2**	(MH "developmental Disabilities")
**S3**	(MH "Mentally Disabled Persons")
**S4**	(mental* or intell*) N3 (impair* or retard* or disab* or defici* or handicap* or subnormal* or sub-normal* or "below normal" or "below average")
**S5**	Learning* N3 (impair* or disab*)
**S6**	S1 OR S2 OR S3 OR S4 OR S5

**S7**	(MH “Exercise*”)
**S8**	(MH “Physical Activity”)
**S9**	(MH “Fitness Centres”)
**S10**	(MH “Health Promotion*”)
**S11**	(exercis* or physical* activ* or fitness centre* or gym* or leisure centre* or health promot*)
**S12**	S7 OR S8 OR S9 OR S10 OR S11
**S13**	S6 AND S12
**S14**	LIMITERS: Publication year 1995-2016, Language: English, Age groups: all adult.

COCHRANE

#1	mental* disab*:ti,ab,kw (Word variations have been searched)
#2	MeSH descriptor: [Intellectual Disability] explode all trees
#3	MeSH descriptor: [Developmental Disabilities] explode all trees
#4	developmental* disab*:ti,ab,kw (Word variations have been searched)
#5	learning disab*:ti,ab,kw (Word variations have been searched)
#6	mental* retard*:ti,ab,kw (Word variations have been searched)
#7	MeSH descriptor: [Mentally Disabled Persons] explode all trees
#8	mental* handicap*:ti,ab,kw (Word variations have been searched)
#9	intelletual* disab*:ti,ab,kw (Word variations have been searched)
#10	#1 or #2 or #3 or #4 or #5 or #6 or #7 or #8 or #9
#11	MeSH descriptor: [Exercise] explode all trees
#12	physical* activ*:ti,ab,kw (Word variations have been searched)
#13	exercise*:ti,ab,kw (Word variations have been searched)
#14	gym*:ti,ab,kw (Word variations have been searched)
#15	leisure centre:ti,ab,kw (Word variations have been searched)
#16	MeSH descriptor: [Health Promotion] explode all trees
#17	health promot*:ti,ab,kw (Word variations have been searched)
#18	#11 OR #12 OR #13 OR #14 OR #15 OR #16 OR #17
	#10 AND #18
	LIMITERS: Publication Year from 1995 to 2015, Trials only

PSYCINFO

**#1**	Exp Learning Disabilities
**#2**	Exp Intellectual Development Disorder
**#3**	Exp Developmental Disabilities
**#4**	((mental* or intel*) adj3 (impair* or retard* or disab* or defici* or handicap* or subnormal* or sub-normal*)).mp.
**#5**	(learning* adj3 (impair* or disab*)).mp.
**#6**	1 OR 2 OR 3 OR 4 OR 5
**#7**	Exp Physical Activity
**#8**	Exp Exercise/
**#9**	Exp Health Promotion/
**#10**	Gym*.mp.
**#11**	Leisure centre*.mp.
**#12**	(exercis* or physical* activ* or health promot*).mp.
**#13**	7 OR 8 OR 9 OR 10 OR 11 OR 12
**#14**	6 AND 13
**#15**	Limit to (English language and yr=”1995-Current”)
